# Community Perspectives on the Creation of a Hospital-Based Doula Program

**DOI:** 10.1089/heq.2020.0096

**Published:** 2021-09-03

**Authors:** Laura B. Attanasio, Marisa DaCosta, Reva Kleppel, Tiki Govantes, Heather Z. Sankey, Sarah L. Goff

**Affiliations:** ^1^ Department of Health Promotion and Policy, University of Massachusetts Amherst, Amherst, Massachusetts, USA.; ^2^ Department of Obstetrics and Gynecology, University of Massachusetts Medical School-Baystate, Springfield, Massachusetts, USA.

**Keywords:** doulas, childbirth, focus groups

## Abstract

**Objective:** Racial and ethnic inequities in perinatal health outcomes are pervasive. Doula support is an evidence-based practice for improving maternal outcomes. However, women in lower-income populations often do not have access to doulas. This study explored community perspectives on doula care to inform the development of a hospital-based doula program to serve primarily low-income women of color.

**Methods:** Four focus groups and four individual interviews were conducted with: (1) women who were pregnant or parenting a child under age 2 (*n*=20); (2) people who had provided support during a birth in the previous 2 years (*n*=5); and (3) women who had received doula training (*n*=4).

**Results:** Participants had generally positive perceptions of doula services. Many aspects of doula support desired by participants are core to birth doula services. Participants identified ways that doulas could potentially address critical gaps in health care services known to impact outcomes (e.g., continuity of care and advocacy), and provide much-needed support in the postpartum period. Responses also suggested that doula training and hospital-based doula programs may need to be adapted to address population-specific needs (e.g., women with substance use disorder and younger mothers). Novel program suggestions included “on call” informational doulas.

**Conclusions:** Findings suggested that women in racial/ethnic minority and lower income groups may be likely to utilize a hospital-based doula program and identified adaptations to traditional doula care that may be required to best meet the needs of women in groups with higher risk of poor maternal health and birth outcomes.

## Introduction

Racial and ethnic inequities in maternal health and perinatal outcomes in the United States are pervasive.^1,2^ Preterm birth, low birthweight, cesarean delivery, and severe maternal morbidity and mortality, are more common among Black, Latina, and Indigenous women than among White women.^1,3–7^ Services from a doula, a professional who provides physical, emotional, and informational support to women and families in the perinatal period, have the potential to reduce inequities in maternal health care and outcomes.^8^ Doula support is associated with positive birth outcomes, including reduction in cesarean delivery rates, decreased use of pain medication, and greater satisfaction with birth experience.^9^ Fewer studies have examined the potential benefits of extended postpartum doula services, but postpartum doula care could impact maternal mental health, maternal/infant bonding,^10–12^ and provide connection to community and medical resources.

Only 6% of U.S. women report having labor support from a doula.^13^ The lack of insurance reimbursement for doula services has resulted in limited access for women in lower-income populations, who are disproportionately women of color.^6,14^ However, when women in these populations do receive doula services, there are substantial benefits, including lower rates of cesarean delivery, preterm birth, and low birth weight.^15,16^ The community-based doula model has been developed to focus on the needs of women of color and low-income women, ideally delivered through organizations led by members of these groups.^17^ Community-based doulas are part of the communities that they work with, and support typically includes more visits and connection to a wider network of services and referrals than the “private pay” doula model.^17^ Community-based doulas can address the lack of racial and socioeconomic diversity in the current doula workforce,^18^ which is important because racially/ethnically and culturally concordant care may foster a positive relationship, including better-quality communication.^19–22^ Women who receive services from community-based doula organizations have increases in breastfeeding duration and decreased chances of cesarean delivery, with larger improvements for Black women.^23,24^

However, community- and hospital-based doula programs have faced challenges, including how to sustain a workforce how to ensure that training and services are tailored to the needs of women in the community.^23,25^ Factors such as cultural perspectives and trust in the health care system may impact use of and experience with doula services.^26^ Although several studies have explored doulas' perspectives on the value of the care they provide,^26–28^ few U.S. studies have explored community members' perceptions of doulas and assessed how previously unmet support needs might be met through doula services. This study examined perspectives on doula care from members of a lower-income community of color with the goal of generating recommendations for a hospital-based doula program.

## Methods

### Setting, study population, and recruitment

This study was conducted in Springfield, MA, a city of ∼150,000, where 48% of the population is Hispanic or Latino, 18% is Black or African American, and 30% has a household income below the federal poverty line.^29^ Springfield has higher rates of preterm birth, low birthweight births, and infant mortality compared with overall rates in Massachusetts.^30^

The following populations were recruited to participate: (1) women who were currently pregnant or had given birth in the previous 2 years, including women in recovery from substance use disorders (SUD); (2) individuals who had provided support during a labor and birth in the previous 2 years; and (3) women who had recently received doula training through a hospital-sponsored initiative in partnership with a community-based doula organization. Recruitment took place at an early childhood education center serving families with lower incomes and a prenatal clinic in a tertiary care teaching hospital. The trained doula focus group was converted to individual video interviews due to COVID restrictions. The study was approved by the Institutional Review Board of Baystate Medical Center.

### Focus groups and interviews

Focus groups and interviews took place February–June 2020. Focus groups were conducted in a private conference room at the early childhood education center by a trained moderator using a semistructured interview guide developed by the research team. The interview guide included questions aimed at understanding community perspectives on doula care, identifying potential unique needs of this population, and eliciting recommendations for doula program implementation (Supplementary Appendix SA1). Each participant received a $50 gift card as compensation for their time. Focus groups and interviews were audiorecorded and professionally transcribed.

### Analysis

Data were analyzed using conventional qualitative content analysis, a form of thematic analysis.^31^ Two members of the research team (L.A. and S.G.) developed a preliminary codebook that consisted of broad categories that were anticipated to emerge in response to each of the interview questions. Three team members (L.A., S.G. and M.D.) performed line-by-line coding on the transcripts, iteratively modifying the codebook as new codes were identified and codes were changed or refined. Each transcript was coded by at least two team members. Coding was reviewed and disagreements resolved through discussion. Codes were then grouped into broader categories to identify emerging themes. An audit trail was created to track coding decisions. The analysis was performed using Dedoose.^32^

## Results

Four focus groups ranging in size from 4 to 9 participants (*N*=23), and 4 individual interviews were conducted, for a total of 27 participants. Focus groups lasted an average of 90 min. Two focus groups included people who were pregnant or recently gave birth, one focus group included people who were pregnant or recently gave birth and identified as being in recovery, and one focus group included support people, two of whom had also recently given birth. Most participants (93%) were female (Table 1). About one-third of participants had education beyond high school. Participants overall were fairly evenly split among White, Black or African American, and Hispanic/Latino/a race/ethnicity. There was, however, some variability in the racial/ethnic composition by focus group (Supplementary Appendix SA2).

**Table 1.** Participant Sociodemographic Characteristics

Characteristic	N	%
Age in years		
Mean (range)	32 (20–72)	
Gender		
Female	25	93%
Male	2	7%
Race/ethnicity^a^		
White	11	41%
Black or African American	8	30%
Hispanic/Latino	11	41%
Education level		
Less than high school	4	15%
High school degree	14	52%
Some college	3	11%
Bachelor's degree or higher	6	22%
Role		
Focus group participants		
Recently gave birth^b^	20	56%
Currently pregnant	5	19%
Support person^b^	5	19%
Recovery^c^	9	33%
Trained doula	4	15%

^a^ One participant identified as both Black and White, and three participants identified as both Hispanic/Latino and White.

^b^ Two participants in the support person focus group had also recently given birth.

^c^ Nine focus group participants identified as being in recovery.

We identified six key themes and associated subthemes; each theme came up across all participant types unless otherwise noted in the text. Two themes were unique to interviews with trained doulas. Themes and illustrative quotes are presented in (Table 2 and Table 3).

**Table 2.** Themes and Illustrative Quotes from Focus Groups and Interviews

Theme	Quotes	Participant role
*1. Awareness and general perceptions of doulas*		
Enthusiasm amid limited prior awareness	“I didn't get told anything about it, and I'm very curious. Because that would have been awesome, I didn't know about it, and I do plan on having one later on so that I would love to know about that and what they do.”	Pregnant or had given birth (recovery group)
	“I didn't really think doulas would like, be there for you like that. So I thought it was mostly like a midwife thing like she was saying… And the way I found out was from someone from [community organization] because she was trying to be a doula. And she needed her certificate, so she was trying to be my doula and I was like, ‘Yeah, sure why not?’”	Pregnant or had given birth
Good for others, not necessarily for me	“My baby is so good, I'm lucky. So I don't think in my opinion it would be helpful for me, but other women, yes. A lot of people have a very hard time adjusting from being just a regular person with not having all this responsibility to them caring for a little human.”	Pregnant or had given birth
Mixed feelings from partners	“I don't know, I think every female has their own different way their pregnancy happens. She had a—it was rough for her, so I don't think me or a person sitting there coaching her, ‘Hey breathe’ is really going help her, you know. I don't know. I guess maybe—I don't know. I really—I don't know, like I said, everyone has their different pregnancies and how they handle it.”	Support person (male)
*2. Doula role*		
Desired scope of work	“After delivery I feel like every mom needs that, because you don't feel yourself. I think I could have used it like afterwards probably through the whole thing, but mainly afterwards because you don't have to feel like yourself, you know like…You don't know who you are before kids…You don't know if you have postpartum because you don't know what the hell it is.”	Pregnant or had given birth (recovery group)
	“Postpartum, somebody who knows postpartum, because I think a lot of us don't understand it”	Pregnant or had given birth (recovery group)
Doula characteristics	“Like what I'm maybe looking for in a doula maybe different than what she needs in the doula.”	Pregnant or had given birth (recovery group)
	“Just basically they put their heart into it, you know what I mean? Whether they got kids or not, just somebody that's there. You know what I mean? Have a real heart for a person.”	Pregnant or had given birth
	“I think culturally appropriate, like someone who is aware of cultures…So there are certain things that…you can do in certain cultures. Like in the Latino culture it's okay if a woman and another woman will like start hugging you or giving you a little more support and touch you. But I know in some other cases it's like, ‘Oh, you're touching me. That's like don't do that.’ That's like a big no. So knowing that culturally appropriate for each culture, that's very, very important, and that's going to break it or make it.”	Support person (female)
Benefits of doula vs. family support	“So, you know, just having a non-biased person available and not connected.”	Pregnant or had given birth
*3. Unmet support needs that doulas could address*		
Core elements of usual doula services	“I think a doula would be really helpful after you have a baby because you're so emotional still, so it's like that's when postpartum can come in. If you don't have that support system there it's really hard. So I think having someone to talk to would be really helpful.”	Pregnant or had given birth
	And she would have been able to explain that to her, what's going on. What does Pitocin do?	Pregnant or had given birth (recovery group)
	“I think it would have been nice to have someone there to, I guess, coach me through all the new experiences I was going through.”	Pregnant or had given birth
Gaps left by the health care system	“I go to [clinic] for my midwife's appointment, and every time it's a different person, it's never the same person. So, they just look at my paper, you know see the notes. There's never, I mean like consistency, support.”	Pregnant or had given birth (recovery group)
	“I remember having the conversation with her and telling her, ‘Why didn't they take the baby and let you sleep?’ And she's like, “I don't know.”…And I, with my own things and my own craziness I didn't—wasn't there for her. I think a doula who would have been there will be like—maybe you should advocate for that point and be like, “You should rest before you go home, because this is a time that you have for resting.”	Support person (male)
Population-specific support needs	“I think that they should have an understanding of the DCF process, because I know that that's a big thing for a lot of women, including myself because I'm going through DCF now. And man, it can be confusing. And I think that having somebody who truly understands what's happening would be great.”	Pregnant or had given birth (recovery group)
*4. Program recommendations*		
Priority populations	“And I know a lot of people that are in shelters or either in different places and they don't have a lot of resources and I think that would be so helpful for mothers that are pregnant and for first time mothers too.”	Pregnant or had given birth (recovery group)
Matching and choice	“Like maybe meet this person on a one on one basic and just find out if their circumstances fit you. You know what I mean?”	Pregnant or had given birth
	Another thing that I also think that is great is if the client has a choice to who her doula is. I can't just come to somebody's door and say, “okay, here, I'm your doula.” And we don't click, and we don't connect.	Trained doula
Connecting women to a doula program	“But I think it definitely should be in the practice. Like for my first appointment when they do the pre-assessment thing, it would have been great to say hey, here's your options you know for a doula as well as the 10, 11 week down syndrome test, like giving you all that information.”	Pregnant or had given birth
New models of doula services	“Maybe most people want to establish a relationship just with one person, but heck, if you've got a question and your person isn't available, might be nice to be able to text somebody at least.”	Pregnant or had given birth

DCF, Department of Children and Families.

**Table 3.** Themes and Illustrative Quotes Unique to Doula Interviews

Theme	Quotes
*5. Barriers and facilitators to becoming or working as a doula*	“The challenges right off were not knowing where to get participants from. Just the people that worked with healthy families kind of know we can get them from where we worked. We didn't know what to do, what direction to go in to, who to talk to about it. It was kind of scary and a lot of doulas that did not pursue to get their certification, I feel was because they had no direction.”
*6. Interactions of doulas and health care personnel*	“I felt that the nurses were not warm and they were not helpful. I felt that they just kind of looked at me…as a person that was not experienced, not as a person who was trying to get the experience.”
	“The one that OB-GYN at [hospital], it was as if they didn't want to recognize me or acknowledge me. The nurses were really hyped that I were there and they were really excited, and they let me have my leeway and if I have questions or if they had questions, we were in it together, but when the OB-GYN came in, it was just like I didn't want nothing to do with me, or I was in their way or—it was really discouraging when it came to the OB-GYN.”

### Theme 1: awareness and general perceptions of doulas

This theme included participants' prior awareness and perceptions of doulas, including general attitudes toward doulas and discussion of the circumstances where doulas might be helpful or not.

#### Enthusiasm amid limited prior awareness

Many participants had heard of doulas, although few had worked with one. There was some confusion about what kind of things is appropriate for doulas to do as part of that role. However, mothers overall had a positive reaction to the idea of doula support, generally endorsing the idea of having more support available. Several expressed a wish to have known about or had access to a doula in their recent birth. This subtheme did not come up in the interviews with trained doulas.

#### Good for others, not necessarily for me

A few participants did not see a benefit to doula support to them personally, although they felt that other women might benefit from doula care. Doula support was perceived as being more necessary for women without partner or family support, or with other challenging circumstances such as a fussier baby. Additionally, focus group participants were unaware of ways that doulas might be helpful during different kinds of births, particularly cesarean births. For women who had heard of doulas before, many had perceived doulas as working with women who were not like them, such as women who were wealthy and/or having a home or water birth. However, participants identified few specific downsides to working with a doula. This subtheme came up across participant types in the focus groups, but not in the interviews with trained doulas.

#### Mixed feelings from partners

Participants who had been a support person at a birth had more mixed feelings about the idea of doulas, and this subtheme was specific to that focus group. Several of them felt that they had provided all of the support that was needed for the person whose birth they attended and believed that having the close relationship of being a family member enabled them to better understand the birthing person's needs and provide comfort.

### Theme 2: doula role

This theme included general views on the desired role of a doula.

#### Desired scope of work

Participants emphasized the need for support during pregnancy and in the postpartum months. Labor support was also seen as positive, but was less of an identified need. Participants noted a need to identify and discuss timing and frequency of visits and to negotiate expectations to avoid a mismatch of expectations. This subtheme did not arise in the interviews with trained doulas.

#### Doula characteristics

Participants felt that people's wishes would vary in terms of having shared life experiences with a doula. For example, some women might want a doula who had given birth herself, while that would be unimportant to others. However, being caring, kind, and nonjudgmental were seen as essential traits. Additionally, participants mentioned the importance of a doula being aware of different cultures and how culture may impact preferences in the perinatal period.

#### Benefits of doula versus family support

Participants noted potential benefits of doula support compared with family support. Family members may bring their own expectations, judgments, and histories, whereas doulas are more “neutral” and familiar and comfortable with birth. Additionally, doulas are there for the birthing person specifically.

### Theme 3: unmet support needs that doulas could address

This theme included discussions of specific support needs that were currently lacking or insufficiently met that could potentially be met through doula services.

#### Core elements of usual doula services

Many aspects of doula support that participants desired are core elements of usual doula services, including emotional support, awareness of mental health needs, material support, providing information and guidance, and supporting family members or partners at the birth.

#### Gaps left by the health care system

Doulas can provide continuity of care throughout the perinatal period, which is not usually possible in the health care system, where women often see different clinicians throughout pregnancy and have typically never met the nurses that will work with them during labor. Advocacy within the health care system was also seen as a key role of doulas. Participants shared experiences in which their symptoms or pain were brushed off or minimized by clinicians. Women in the SUD-specific focus group related negative experiences of treatment in the hospital after giving birth, such as nurses expressing skepticism about whether a urine test would detect drugs, and noted the potential benefits of doula advocacy in such situations.

#### Population-specific support needs

Focus group respondents identified specific populations that might also have particular support needs, such as women with lower incomes, young mothers, first-time mothers, and women with SUD. Young mothers and first-time mothers were perceived as needing more informational and emotional support than older women or women who had given birth before, and women with lower incomes were seen as potentially benefitting from connection to locations or organizations that could provide material resources such as free diapers. Women with SUD may be involved with the Department of Children and Families, and desired support in navigating the bureaucracy from someone who is on “your side.” Women with SUD also identified concerns with how to manage taking pain medications after a cesarean birth and the impact on recovery. Nonjudgmental doula support was desired, “because that's something that you don't really want to talk to your doctor about.”

### Theme 4: program recommendations

Many of the specific recommendations for a hospital-based doula program are connected to the previous themes.

#### Priority populations

Participants were enthusiastic about the idea of doulas being available to all who want doula services, but also noted specific populations that could potentially be prioritized if there were not enough resources to make doulas available to all. These populations overlapped with the population-specific support need identified in Theme 3, and included first-time moms, young moms, women who lacked other support, and women with SUD.

#### Matching and choice

Given the perceived variation in what might be important to women in choosing a doula (Theme 2), participants believed that a system should be created to match women with doulas and to allow women some choice of doulas, so that each individual woman could prioritize the characteristics that she felt were important in a doula. Some women felt that ideally there would be an opportunity for a woman and a doula to interact to see if they connected well in terms of personality, beyond attributes that could be identified through a questionnaire.

#### New models of doula services

Participants suggested new models of doula services that might be beneficial, including doulas who only provide phone or text support during pregnancy. Some participants also noted the overlooked needs of new fathers, and proposed “doula dads,” or experienced fathers/partners whose primary role would be to support the partner. This subtheme came up only in the support person focus group and in the interviews with trained doulas.

#### Connecting women to a doula program

Participants felt that for a new doula program to be successful, there would need to be efforts to inform women about the role of a doula and the ways that doulas can provide support for women in a range of circumstances. Most participants felt that it would be helpful if women were informed about and connected to the doula program through their prenatal care provider. Others felt that other places in the community that serve women in lower-income populations, such as the office for the Special Supplemental Nutrition Program for Women, Infants and Children (WIC).

### Themes unique to doula interviews

#### Theme 5: barriers and facilitators to becoming or working as a doula

Challenges associated with participating in doula training included finding a training program that addressed desired skills, and accessibility in terms of location and cost (Table 3). Doulas also discussed challenges with transitioning to work as a doula after training, including finding clients and attending first births without having had the opportunity to apprentice with an experienced doula. Employment could be a barrier or facilitator; some trained doulas had jobs that were compatible with attending births, or even helped them to find clients because they worked with pregnant women. For others, it was difficult or impossible to negotiate being on call with their employment.

#### Theme 6: interactions of doula and health care personnel

Interactions with health care providers, including nurses, physicians, and midwives, were an important aspect of doulas' experiences at births they had attended. Participants mentioned both situations where they were made to feel welcome and valued, and situations where they felt in the way or even felt hostility from health care providers. Some participants perceived that the environments that were less welcoming to doulas might be where advocacy was most needed. Participants discussed the importance of getting the buy-in of clinicians at all levels for the success of a hospital-based doula program.

## Discussion

Many of the desired services identified by women in this study were consistent with a traditional privately-paid doula's role. Some of the potential benefits of extending doula support to this population would likely arise simply by making this successful model of support accessible to more women. However, we also found some differences in emphasis as well as unique needs for women in the community studied, compared with privately paid doula services.

Notably, participants emphasized support needs during pregnancy and the postpartum period over and above support during labor and birth. However, they did also identify additional ways that doulas could be helpful during labor. Interestingly, one specific example that arose was that a doula could help advocate for admission to the hospital, which seems to contradict one mechanism by which doula care may lead to lower intervention—delaying admission to the hospital until active labor. This seeming contradiction may reflect the difficulty in fully conceptualizing doula support among women who, for the most part, had not experienced it personally. It could also reflect a lack of knowledge about the labor process and the potential benefits of laboring at home for longer, which doula support could also address. The postpartum period was seen as a time when women's needs are extensive and family support might not be adequate, especially given the high prevalence and under-recognition of postpartum depression. The American College of Obstetricians and Gynecologists has new guidelines highlighting the importance of the postpartum period for maternal wellbeing^33^; our findings indicate that doulas could be an important part of the postpartum support system.

Participants also identified ways in which doulas could help to address gaps or negative experiences in the health care system, through continuity of care and advocacy. Discrimination and disrespectful treatment are reported by up to 24% of birthing people, and may be particularly prevalent among women of color^34–40^; doula advocacy could help buffer the effects of such treatment.^26^ However, to optimally integrate doulas into a hospital-based program in an advocacy role, institutional commitments beyond the addition of doula services may be necessary. The doulas that we interviewed highlighted the importance of their interactions with clinicians in making them feel welcome or discouraged. Previous research has shown that clinicians may have negative perceptions of doulas if they have experiences interacting with them in an adversarial manner.^41,42^ A recent survey of maternity care clinicians suggested that educating clinicians about doulas' training and creating clear guidelines for the role of doulas within the maternity care team could foster positive doula–clinician relationships.^41^

The support needs identified by participants largely mirrored the model of community-based doula programs, which are designed to address the needs of underserved communities. While this study explored the creation of a hospital-based doula program, a community-based doula approach has successfully been integrated into some hospital-based doula programs.^43^ For doulas to deliver this enhanced support, the allocation of adequate resources is necessary, and doulas must be fairly compensated for the work involved to avoid burnout and to promote sustainability^26^; a hospital-based program must also take into account these resource considerations.

The focus group with women in recovery identified several ways in which tailored doula support could be particularly beneficial for this population. Stigmatization of patients with SUD is a general problem, and is particularly acute when the patient is a pregnant woman,^44–46^ underscoring the importance of advocacy and the potential role of a doula for these women, as our focus group participants noted. This is very salient in the context of Springfield, where opioid-related deaths dramatically increased in recent years even as statewide opioid-related deaths fell.^47^

### Strengths and limitations

Unique contributions of this study include examining partners' perceptions of doulas, an identified gap in doula research,^48^ and exploring women's own perspectives of their support needs in the perinatal period and the role of doulas in addressing them. However, there are some limitations to consider. In developing the focus group guide, we did not solicit input from currently practicing doulas or members of the participant populations. It is possible that the definition of doula services influenced participant responses. Additionally, we purposefully asked open-ended questions about perinatal experiences to allow women to construct their own experience narratives, but the lack of explicit questions about mistreatment, disparities in treatment, and culture-matched care may also have influenced responses. We only included English-speaking women; a Spanish-speaking focus group was scheduled, but canceled due to COVID. Perceptions of doulas held by maternity care clinicians may be important,^41^ but was beyond the scope of this study. While the focus group moderators encouraged an atmosphere conducive to open discussion, it is possible that responses were influenced by social desirability bias.

## Conclusions

Findings suggested that women in racial/ethnic minority and lower income groups may be likely to utilize a hospital-based doula program and identified adaptations to traditional doula care that may be required to best meet the needs of women in groups with higher risk of poor maternal health and birth outcomes.

## Supplementary Material

Supplemental data

Supplemental data

## Supplementary Material

Supplementary Appendix SA1

Supplementary Appendix SA2

## Abbreviations Used

DCF: Department of Children and Families
SUD: substance use disorders

## References

[1] Bryant AS, Worjoloh A, Caughey AB, et al. Racial/ethnic disparities in obstetric outcomes and care: prevalence and determinants. Am J Obstet Gynecol. 2010;202:335–343. 20060513 10.1016/j.ajog.2009.10.864 PMC2847630

[2] Howell EA, Brown H, Brumley J, et al. Reduction of peripartum racial and ethnic disparities: a conceptual framework and maternal safety consensus bundle. Obstet Gynecol. 2018;131:770–782. 29683895 10.1097/AOG.0000000000002475

[3] Howell EA, Zeitlin J. Improving hospital quality to reduce disparities in severe maternal morbidity and mortality. Semin Perinatol. 2017;41:266–272. 28735811 10.1053/j.semperi.2017.04.002 PMC5592149

[4] Admon LK, Winkelman TNA, Zivin K, et al. Racial and ethnic disparities in the incidence of severe maternal morbidity in the United States, 2012–2015. Obstet Gynecol. 2018;132:1158–1166. 30303912 10.1097/AOG.0000000000002937

[5] Leonard SA, Main EK, Scott KA, et al. Racial and ethnic disparities in severe maternal morbidity prevalence and trends. Ann Epidemiol. 2019;33:30–36. 30928320 10.1016/j.annepidem.2019.02.007 PMC6502679

[6] Martin JA, Hamilton BE, Osterman MJK, et al. Births: final Data for 2018. Natl Vital Stat Rep. 2019;68:1–104. 32501202

[7] Kozhimannil KB, Interrante JD, Tofte AN, et al. Severe maternal morbidity and mortality among indigenous women in the United States. Obstet Gynecol. 2020;135:294–300. 31923072 10.1097/AOG.0000000000003647 PMC7012336

[8] DONA International. What is a doula? Available at www.dona.org/what-is-a-doula Accessed April 20, 2019.

[9] Bohren MA, Hofmeyr GJ, Sakala C, et al. Continuous support for women during childbirth. Cochrane Database Syst Rev. 2017. DOI: 10.1002/14651858.CD003766.pub6. PMC6483123 28681500

[10] McComish JF, Visger JM. Domains of postpartum doula care and maternal responsiveness and competence. J Obstet Gynecol Neonatal Nurs. 2009;38:148–156. 10.1111/j.1552-6909.2009.01002.x 19323711

[11] Campbell-Voytal K, Fry McComish J, Visger JM, et al. Postpartum doulas: motivations and perceptions of practice. Midwifery. 2011;27:e214–e221. 21055853 10.1016/j.midw.2010.09.006 PMC3048903

[12] Gjerdingen DK, McGovern P, Pratt R, et al. Postpartum doula and peer telephone support for postpartum depression: a pilot randomized controlled trial. J Prim Care Community Health. 2013;4:36–43. 23799688 10.1177/2150131912451598

[13] Declercq E, Sakala C, Corry M, et al. Listening to Mothers III: Pregnancy and Childbirth. New York, NY: Childbirth Connection, 2013.

[14] Semega J, Kollar M, Shrider EA, et al. Income and Poverty in the United States: 2019. Washington, DC: US Census Bureau, 2020.

[15] Thomas M-P, Ammann G, Brazier E, et al. Doula services within a healthy start program: increasing access for an underserved population. Matern Child Health J. 2017;21(S1):59–64. 29198051 10.1007/s10995-017-2402-0 PMC5736765

[16] Kozhimannil KB, Hardeman RR, Attanasio LB, et al. Doula care, birth outcomes, and costs among Medicaid beneficiaries. Am J Public Health. 2013;103:e113–e121. 23409910 10.2105/AJPH.2012.301201 PMC3617571

[17] Bey A, Brill A, Porchia-Albert C, et al. Advancing birth justice: community-based doula models as a standard of care for ending racial disparities. New York, NY: Every Mother Counts, 2019. Available at: https://everymothercounts.org/wp-content/uploads/2019/03/Advancing-Birth-Justice-CBD-Models-as-Std-of-Care-3-25-19.pdf Accessed September 15, 2020.

[18] Lantz PM, Low LK, Varkey S, et al. Doulas as childbirth paraprofessionals: results from a national survey. Women's Health Issues. 2005;15:109–116. 15894196 10.1016/j.whi.2005.01.002

[19] Chu J, Wang N, Choi YS, et al. The effect of patient-centered communication and racial concordant care on care satisfaction among U.S. immigrants. Med Care Res Rev. 2019. [Epub ahead of print]; DOI: 10.1177/1077558719890988. 31793385

[20] Shen MJ, Peterson EB, Costas-Muniz R, et al. The effects of race and racial concordance on patient-physician communication: a systematic review of the literature. J Racial Ethn Health Disparities. 2018;5:117–140. 28275996 10.1007/s40615-017-0350-4 PMC5591056

[21] Cooper LA, Roter DL, Johnson RL, et al. Patient-centered communication, ratings of care, and concordance of patient and physician race. Ann Intern Med. 2003;139:907–915. 14644893 10.7326/0003-4819-139-11-200312020-00009

[22] Greenwood BN, Hardeman RR, Huang L, et al. Physician-patient racial concordance and disparities in birthing mortality for newborns. Proc Natl Acad Sci U S A. 2020;117:21194–21200. 32817561 10.1073/pnas.1913405117 PMC7474610

[23] Bakst C, Moore JE, George KE, et al. Community-Based Maternal Support Services: The Role of Doulas and Community Health Workers in Medicaid. Washington, DC: Institute for Medicaid Innovation, 2020

[24] HealthConnect One. The Perinatal Revolution. Chicago, IL, 2014.

[25] Kozhimannil KB, Vogelsang CA, Hardeman RR. Medicaid Coverage of Doula Services in Minnesota: Preliminary Findings from the First Year. 2015.

[26] Wint K, Elias TI, Mendez G, et al. Experiences of community doulas working with low-income, African American mothers. Health Equity. 2019;3:109–116. 31289769 10.1089/heq.2018.0045 PMC6608698

[27] Hardeman RR, Kozhimannil KB. Motivations for entering the doula profession: perspectives from women of color. J Midwifery Women's Health. 2016;61:773–780. 27862907 10.1111/jmwh.12497 PMC5143171

[28] Gilliland AL. After praise and encouragement: emotional support strategies used by birth doulas in the USA and Canada. Midwifery. 2011;27:525–531. 20850916 10.1016/j.midw.2010.04.006

[29] Census Reporter: Springfield, MA. Available at: https://censusreporter.org/profiles/16000US2567000-springfield-ma Accessed September 3, 2020.

[30] Szegda K, Collins J, Hudson S. Springfield Health Equity Report: Looking at Health through Race and Ethnicity, 2019 Update. Springfield, MA, 2019. Available at: http://www.euro.who.int/__data/assets/pdf_file/0018/103824/E89384.pdf%0A http://www.countyhealthrankings.org Accessed September 7, 2020.

[31] Hsieh H-F, Shannon SE. Three approaches to qualitative content analysis. Qual Health Res. 2005;15:1277–1288. 16204405 10.1177/1049732305276687

[32] Dedoose Version 8.0.35, web application for managing, analyzing, and presenting qualitative and mixed method research data. 2018. Available at: www.dedoose.com

[33] Presidential Task Force of Redefining the Postpartum Visit, Committee on Obstetric Practice. ACOG Committee Opinion No. 736: optimizing Postpartum Care. Obstet Gynecol. 2018;131:e140–e150. 29683911 10.1097/AOG.0000000000002633

[34] Attanasio L, Kozhimannil KB. Patient-reported communication quality and perceived discrimination in maternity care. Med Care. 2015;53:863–871. 26340663 10.1097/MLR.0000000000000411 PMC4570858

[35] Attanasio LB, Hardeman RR. Declined care and discrimination during the childbirth hospitalization. Soc Sci Med. 2019;232:270–277. 31112918 10.1016/j.socscimed.2019.05.008

[36] McLemore MR, Altman MR, Cooper N, et al. Health care experiences of pregnant, birthing and postnatal women of color at risk for preterm birth. Soc Sci Med. 2018;201:127–135. 29494846 10.1016/j.socscimed.2018.02.013

[37] Ertel KA, James-Todd T, Kleinman K, et al. Racial discrimination, response to unfair treatment, and depressive symptoms among pregnant black and African American women in the United States. Ann Epidemiol. 2012;22:840–846. 23123506 10.1016/j.annepidem.2012.10.001 PMC4643652

[38] Nuru-Jeter A, Dominguez TP, Hammond WP, et al. “It's the skin you're in”: African-American women talk about their experiences of racism. An exploratory study to develop measures of racism for birth outcome studies. Matern Child Health J. 2009;13:29–39. 18463971 10.1007/s10995-008-0357-x PMC3051354

[39] Altman MR, Oseguera T, McLemore MR, et al. Information and power: women of color's experiences interacting with health care providers in pregnancy and birth. Soc Sci Med. 2019;238:112491. 31434029 10.1016/j.socscimed.2019.112491

[40] De Marco M, Thorburn S, Zhao W. Perceived discrimination during prenatal care, labor, and delivery: an examination of data from the Oregon Pregnancy Risk Assessment Monitoring System, 1998–1999, 2000, and 2001. Am J Public Health. 2008;98:1818–1822. 18703444 10.2105/AJPH.2007.123687 PMC2636464

[41] Neel K, Goldman R, Marte D, et al. Hospital-based maternity care practitioners' perceptions of doulas. Birth. 2019:1–7. [Epub ahead of print]; DOI: 10.1111/birt.12420. 30734958

[42] Torres JMC. Breast milk and labour support: lactation consultants' and doulas' strategies for navigating the medical context of maternity care. Sociol Health Illn. 2013;35:924–938. 23398567 10.1111/1467-9566.12010

[43] Mottl-Santiago J, Herr K, Rodrigues D, et al. The Birth Sisters program: a model of hospital-based doula support to promote health equity. J Health Care Poor Underserved. 2020;31:43–55. 32037316 10.1353/hpu.2020.0007

[44] Kroelinger CD, Rice ME, Cox S, et al. State strategies to address opioid use disorder among pregnant and postpartum women and infants prenatally exposed to substances, including infants with neonatal abstinence syndrome. MMWR Morb Mortal Wkly Rep. 2019;68:777–783. 31513558 10.15585/mmwr.mm6836a1 PMC6753967

[45] Saia KA, Schiff D, Wachman EM, et al. Caring for pregnant women with opioid use disorder in the USA: expanding and improving treatment. Curr Obstet Gynecol Rep. 2016:257–263. [Epub ahead of print]; DOI: 10.1007/s13669-016-0168-9. 27563497 PMC4981621

[46] Stone R. Pregnant women and substance use: fear, stigma and barriers to care. Health Justice. 2015;3:1–15.

[47] Current opioid statistics. Mass.gov. Available at: https://www.mass.gov/lists/current-opioid-statistics Accessed August 23, 2019.

[48] Steel A, Frawley J, Adams J, et al. Trained or professional doulas in the support and care of pregnant and birthing women: a critical integrative review. Health Soc Care Community. 2015;23:225–241. 24942339 10.1111/hsc.12112

