# Supplementary material for: Community Perspectives on the Creation of a Hospital-Based Doula Program
Source: Health Equity. 2021 Sep 3;5(1):545–53. doi: 10.1089/heq.2020.0096 (PMC8665817; doi:10.1089/heq.2020.0096)
Supplement: Supplemental data [file Supp_AppS1.docx]

**Appendix 1. Interview Guide - Mothers**

1. [Warm up] Tell us about one thing that surprised you about pregnancy, delivery, or the after you went home with your new baby.

2. We asked you all to join us today, so we could try to better understand your thoughts, feelings, and perspectives about doulas. So, first we wanted to ask if anyone has heard of doulas before. [If so, probe what the term means to them, the context in which they heard about doulas, what they see as the potential pros and cons of working with them. If any participants have worked with a doula, how they came to work with one, pros and cons of the experience.

3. Doulas can play different roles during a pregnancy, during birth, or after birth. For today, when we talk about a doula, we mean a support person who is not medically trained but who is trained to give women help or support they need during pregnancy, during labor and delivery, and after a woman goes home after delivery.

Some examples of what a doula can do while you are pregnant is give you emotional support and help you get ready for labor and delivery. During birth, they can help you feel more comfortable by rubbing your back or giving you emotional support or helping you talk to the doctor or nurse about what you need. Finally, when you are home after delivery, they can help with breastfeeding if you want, provide emotional support like listening to your feelings, help you out by watching the baby so you can take a nap, and connecting you to resources that may be helpful, like WIC or a breastfeeding group.

A number of research studies have shown that working with a doula during labor and delivery may make women less likely to have a c-section, reduce the need for medication for pain relief, reduce the length of labor, and help you to have a good birth experience.

3a. With that understanding of what a doula can do, can you think about how a doula might have been helpful or not so helpful during your pregnancy?

Probe:

Even if you didn’t have something specific you think they might have helped with, can you talk a little bit about how you might see a doula being potentially helpful or not?

3b. Now, can you think back to the birth of your child and what it might have been like to have a doula there – what might have been helpful or not so helpful?

Probe:

Even if you didn’t have something specific you think they might have helped with, can you talk a little bit about how you might see a doula being potentially helpful or not?

3c. And last, let’s talk about what you see might have been the benefit or lack of benefit to having doula care at home after your delivery. Even if you didn’t have something specific you think they might have helped with, can you talk a little bit about how you might see a doula being potentially helpful or not?

4. Now we’d like to ask for your help in thinking about how to design a doula program at [Hospital]. We would like to understand what information you think is important to give to women about doulas ahead of time.

Doulas aren’t usually covered by insurance, so in a lot of cases they work for private clients who pay them directly. Typically birth doula services are a package that includes about two visits during pregnancy, the doula being present at the labor and birth, and then one or two more visits in the week or two after the baby is born. There is another type of doula called a postpartum doula that focuses on helping moms after the baby is born (those things like helping with breastfeeding, providing emotional support, and watching the baby while you take a nap, that I mentioned a few minute ago). These services are usually provided on an hourly basis.

When considering how to create a program at [Hospital], it wouldn’t necessarily have to look like this, so we would be interested in your perspective on what would be most helpful, such as number of visits and timing.

Probes:

What might make you more or less interested in working with a doula?

Do you think you might be more likely to work with a doula if you and she had shared life experiences? If so, why?

5. Is there anything else you think is important to think about when thinking about creating a program to offer doula care to women?
