# Supplementary material for: Community Perspectives on the Creation of a Hospital-Based Doula Program
Source: Health Equity. 2021 Sep 3;5(1):545–53. doi: 10.1089/heq.2020.0096 (PMC8665817; doi:10.1089/heq.2020.0096)
Supplement: Supplemental data [file Supp_AppS2.docx]

| **Appendix 2. Racial and ethnic composition by focus group.** | | | |  |
| --- | --- | --- | --- | --- |
|  | **Black** | **White** | **Latino/a** | **Black and White** |
| Focus group 1 | 40 | 0 | 60 | 0 |
| Focus group 2 | 0 | 25 | 75 | 0 |
| Focus group 3 (support people) | 22 | 11 | 67 | 0 |
| Focus group 4 (recovery)^1^ | 0 | 67 | 22 | 11 |
| ^1^The racial/ethnic composition of this group reflected the composition of the recovery group at the community partner site for this study. | | | | |
